# Peripheral versus Marrow Lipidomics in Patients with Severe Aplastic Anemia: Potential Indicators for Early Immunosuppressive Treatment Response

**DOI:** 10.1002/lipd.70034

**Published:** 2026-01-19

**Authors:** Zexing Sun, Yilei Hong, Yaonan Hong, Chuanao Xin, Qi Liu, Huijin Hu, Yingying Shen, Ying Chen, Shan Liu, Yiping Shen, Yuhong Zhou, Dijiong Wu

**Affiliations:** ^1^ Department of Hematology The First Affiliated Hospital of Zhejiang Chinese Medical University (Zhejiang Provincial Hospital of Chinese Medicine) Zhejiang Hangzhou China; ^2^ The First School of Clinical Medicine Zhejiang Chinese Medical University Zhejiang Hangzhou China; ^3^ National Traditional Chinese Medicine Clinical Research Base (Hematology) Zhejiang Hangzhou China; ^4^ Department of Clinical Evaluation Center The First Affiliated Hospital of Zhejiang Chinese Medical University Zhejiang Hangzhou China; ^5^ Department of Oncology and Hematology Wenzhou Hospital of Integrated Traditional Chinese and Western Medicine Affiliated to Zhejiang Chinese Medicine University Zhejiang China

**Keywords:** aplastic anemia, early response, immunosuppressive therapy, lipidomics, prediction biomarker

## Abstract

This study aimed to explore the differences of peripheral blood (PB) and bone marrow serum lipidomic profiles in severe aplastic anemia (SAA) patients and their significance in predicting earlier immunosuppressive therapy (IST) response. A cohort of 11 newly diagnosed SAA patients and 15 healthy controls were enrolled between June 2020 and November 2022, and six of the SAA patients received ATG‐based IST. PB and BM serum were collected for further LC–MS/MS analysis. Compared to donors, SAA patients exhibited more pronounced abnormalities in lipid metabolism profiles within BM serum relative to PB serum. Specifically, elevated levels of free fatty acids (FA), fatty acid esters of hydroxyl fatty acids (FAHFA), and phosphatidylserine (PS) were noted in the BM serum of SAA patients. Following treatment, there was a noted increase in acylcarnitine (ACar), hexosylceramide non‐hydroxy fatty acid‐sphingosine (HexCer‐NS), and sulfur hexosyl ceramide hydroxy fatty acid (SHexCer), while levels of lysophosphatidylcholine (LPC) and oxidized phosphatidylcholine (OxPC) diminished, particularly in complete or partial response (CR/PR) patients. Acknowledging the changes of BM lipidomics may contribute to earlier prediction of ATG‐based IST response in SAA patients.

## 1 Introduction

Aplastic anemia (AA) is a relatively uncommon hematological disorder characterized by the presence of pancytopenia, which is attributed to bone marrow (BM) failure (Red Blood Cell Disease Group 2022). According to the disease severity, patients can be separated into severe aplastic anemia (SAA) and non‐severe aplastic anemia (NSAA) (Camitta et al. 1975). The development of AA has not yet been fully illustrated. Immune disorders, abnormal hematopoietic microenvironment, and defects of hematopoietic stem and progenitor cells all contribute to the progression of AA (Wang and Liu 2019). Among these, the research on immune abnormalities and T cell hyperfunction is the most comprehensive (Zhu et al. 2021), providing the theoretical basis for immunosuppressive therapy (IST) (Giudice and Selleri 2022). IST with the combination of anti‐thymocyte globulin (ATG) and cyclosporine (CsA) is recommended as the primary first‐line treatment for SAA who were not suitable for stem cell transplantation; however, only 40%–60% of AA patients achieved disease improvement (Afable et al. 2011). To date, many factors such as residual hematopoiesis, age of onset, disease duration, and somatic mutations also hold predictive value (Vaht et al. 2018). However, clinically, we mostly need to wait for the median response time of around 6 months to make a further decision on whether the patients need repeated intensity IST or salvage hematopoietic stem cell transplantation (HSCT). Hence, for patients prepared for IST, additional measures are necessary to anticipate treatment efficacy at an earlier stage.

While traditional lipid biomarkers including low‐density lipoprotein cholesterol (LDL‐C), high‐density lipoprotein cholesterol (HDL‐C), and triglycerides (TG) are well‐established contributors to cardiovascular pathogenesis via pro‐inflammatory activation and cellular metabolic dysregulation (Ference et al. 2018; Soppert et al. 2020), emerging lipidomic profiling has identified novel lipid species as critical regulators in the diagnosis, prognostic stratification, and personalized therapeutic strategies for multifactorial diseases. For instance, phosphatidylethanolamine (PE), phosphatidylcholine (PC), and ceramides demonstrated superior diagnostic performance, whereas elevated monohexosylceramide levels were associated with good prognosis in malignant mesothelioma (Chen et al. 2022). In addition, Liang et al. (Liang et al. 2023) identified distinct spectral signatures in lipid metabolic markers through laser Raman spectroscopy combined with OPLS‐DA in serum samples from AA and MDS patients, providing new spectroscopic evidence for the non‐invasive differential diagnosis of bone marrow failure disorders. Ruan J et al. (Ruan et al. 2023) found that lipid profiles of patients' PB (peripheral blood) serum not only help distinguish between transfusion‐dependent NSAA and hypocellular myelodysplastic syndrome (h‐MDS) but also predict the different CsA responses. Also, our previous study on PB serum revealed a negative correlation between the baseline apolipoprotein‐A (Apo‐A) level and 9‐month responses, indicating a prognostic predictor for SAA or very severe AA under ATG‐based IST (Liu et al. 2022).

However, lipid metabolism in the PB may not entirely elucidate alterations in BM. In AA patients, the inclination of BM mesenchymal stem cells (MSCs) to differentiate toward adipocytes may contribute to abnormal lipid metabolism, thereby impacting the function of hematopoietic stem cells (HSCs) (Wang and Liu 2019). Application of lipidomics can aid in elucidating the difference in lipid metabolism between BM serum and PB serum, as well as changes before and after IST, thus laying a preliminary foundation for further in‐depth research. Through this method, this study aimed to reveal the difference in sample derivation and lipid metabolism before and after ATG‐based IST, offering new insights into microenvironment changes underlining IST treatment.

## 2 Patients and Methods

### 2.1 Patients

The study was prospectively registered at chictr.org.cn as # ChiCTR2100052979. Eleven newly diagnosed SAA patients at Zhejiang Provincial Hospital of Traditional Chinese Medicine were enrolled in this study between June 2020 and December 2022. The inclusion criteria were as follows: (1) diagnosing SAA according to the Camitta criteria and not receiving other immunosuppressant treatment before (Red Blood Cell Disease Group 2022); (2) not participating in other clinical research; (3) 6 < age ≤ 50 years old; (4) informed consent. The exclusion criteria were: (1) patients with severe organ dysfunction, metabolic disorders, and mental disorders; (2) patients with hyperlipidemia, fatty liver, emaciation, or overweight; (3) pregnant or preparing for pregnancy, or lactating women; (4) other diseases that could cause pancytopenia, including MDS, paroxysmal nocturnal hemoglobinuria, and malignancies.

### 2.2 Selection of Healthy Donors

Healthy donors for HSCT who underwent a physical examination during the same period were selected as controls. The inclusion criteria were as follows: (1) body mass index (BMI) for adult donors: 18.5–23.9 (kg/m^2^) (Chinese Nutrition Society Obesity Prevention and Control Section 2022); BMI for the minors was determined according to the Chinese standard; (2) no history of hyperlipidemia or fatty liver; (3) no history of other metabolic or endocrine diseases.

### 2.3 Clinical Data Collection

The general conditions at baseline of both patients and donors, including age, sex, height, weight, BMI, white blood cell (WBC) count, absolute neutrophil count (ANC), hemoglobin (HGB), platelet count (PLT), percentage of reticulocytes (Ret %), and BM hyperplasia grade were recorded. Patients who underwent IST had their complete blood count monitored every 3 days (adjust the interval as needed) for up to 9 months after IST, with BM serum reevaluations conducted 3 months post‐treatment.

### 2.4 Treatments

Patients who underwent IST all received ATG and CsA. Rabbit ATG (r‐ATG, Sangstat, California, United States) was administered for 5 days (with a daily dose of 3–5 mg/kg); CsA was given at the dose of 3–6 mg/kg × d^−1^ with a dosing frequency of q12 h, and the dosage was adjusted to maintain the trough concentration at 150–250 ng/L.

### 2.5 Response Criteria

The therapeutic response was evaluated 9 months following the initiation of IST according to the following criteria: (1) complete response (CR): levels of HGB reached normal values (120 g/L for male and 110 g/L for female), ANC above 1.5 × 10^9^/L and PLT above 100 × 10^9^/L; (2) partial response (PR): (a) transfusion independent; (b) HGB increased > 30 g/L compared with the baseline and was maintained for at least 3 months; (3) no response (NR): still met the diagnostic criteria for SAA (Red Blood Cell Disease Group 2022, 2017).

### 2.6 Grouping

This study provides a comprehensive analysis of lipid metabolism profiles by comparing SAA patients (P) with healthy donors (D) in both PB and BM serum, delineating them into two comparative groups (P_PB_ vs. D_PB_ and P_BM_ vs. D_BM_). Additionally, the differences in lipid metabolism profiles between PB and BM serum within SAA patients (P) were assessed (P_PB_ vs. P_BM_). The pre‐ and post‐interventional analysis primarily concentrates on BM serum. Utilizing donors' BM serum (D_BM_) as a control, the study evaluated alterations in BM lipid metabolism profiles in patients achieving CR/PR (D_BM_ vs. CR/PR_BM‐pre_ and D_BM_ vs. CR/PR _BM‐post_) alongside those classified as non‐responders (D_BM_ vs. NR_BM‐pre_ and D_BM_ vs. NR_BM‐post_).

### 2.7 Liquid Chromatography–Tandem Mass Spectrometry (LC–MS/MS) Analysis

#### 2.7.1 Collection of Specimens

Collecting 5 mL fasting PB serum and 5 mL BM serum samples from SAA patients and healthy donors at baseline. Three months after IST, fasting PB and BM serum samples were gathered from patients who received IST. After standing upright at room temperature for 1 h, specimens were transferred into a centrifuge and spun at 3000 rpm for 10 min. The supernatants were then extracted and stored in EP tubes (1.5 mL) and further frozen (−80°C).

#### 2.7.2 Metabolites Extraction

A 100 μL sample was transferred into a 480 μL extract solution containing 20% methanol in MTBE. Vortexed for 30 s, the resulting mixture was then sonicated in the ice water for 10 min. Subsequently, samples were placed in an incubator at −40°C for another 1 h. In further, samples were centrifugated with 3000 rpm for 15 min (at 4°C). Following this, 350 μL supernatant was collected and dried at 37°C in a vacuum concentrator. To the next, the samples were further reconstituted in a 100 μL solution of dichloromethane and methanol in a 1:1 ratio (30 s). After sonication in the ice‐water bath (10 min), the samples were transferred for centrifugation with 12,000 rpm at 15 min (4°C). Eventually, 80 μL of samples was used for final analysis (LC/MS), and a mixture of 20 μL of the supernatants from each sample were prepared for quality control (QC) assessment. These QC samples were injected at regular intervals throughout the analytical run to monitor instrument stability. The tight clustering of QC samples in the PCA score plot (Figure S1) and the low relative standard deviation (RSD) of internal standards confirmed the high reproducibility and stability of the system during the analysis.

#### 2.7.3 LC–MS/MS Analysis

For the LC–MS/MS analysis, an ultra‐high performance liquid chromatography (UHPLC) system (Vanquish, Thermo Fisher Scientific, Massachusetts, United States) and a Waters ACQUITY UPLC HSS T3 liquid column were utilized. The Thermo Q Exactive HFX mass spectrometer utilized in this study operates with a full MS resolution of 120,000 and an MS/MS resolution of 7500. The mobile phase A (60% acetonitrile mixture with 40% water) and mobile phase B (included 10% acetonitrile mixture with 90% isopropanol) were prepared, which were further supplemented with 10 mM ammonium formate. The elution protocol was as follows: 0–1.0 min, 40% B; 1.0–12.0 min, 40%–100% B; 12.0–13.5 min, 100% B; 13.5–13.7 min, 100% ~ 40% B; 13.7–18.0 min, and 40% B. The flow rate of the mobile phase was set at 0.3 mL/min, the temperature of the column was set at 55°C, the temperature of the auto‐sampler was set at 4°C, and the injection volume at 2 μL (positive) or 2 μL (negative). Utilizing the Q Exactive HFX mass spectrometer (Orbitrap MS, Thermo Fisher Scientific, Massachusetts, United States) in the data‐dependent acquisition mode controlled by the Xcalibur 4.0.27 acquisition software (Thermo Fisher Scientific, Massachusetts, United States). Configured ESI source conditions as follows: Sheath gas flow rate: 30 Arb, Auxiliary gas flow rate: 10 Arb, Capillary temperature: 350°C, Collision energy: 10/30/60 in NCE mode, Spray Voltage: 4 kV (positive) or −3.8 kV (negative).

#### 2.7.4 Data Processing Methodology

##### 2.7.4.1 Raw Data Conversion

Raw mass spectrometry data were converted to the mzXML format using ProteoWizard software to facilitate downstream processing.

##### 2.7.4.2 Data Preprocessing

Data preprocessing involved several critical steps to enhance data quality and reliability for subsequent analyses. First, outlier filtering was employed to reduce noise by filtering peaks based on RSD. Following this, missing value filtering was conducted, wherein peaks with missing values exceeding 50% in any group or across all groups were excluded from the analysis. For the remaining missing values, imputation was performed using half of the minimum detected value to ensure no significant information was lost. Additionally, internal standards were applied to normalize peak area data, further refining the dataset. Collectively, these preprocessing steps significantly mitigated noise and experimental variability, ensuring a robust foundation for the analyses that followed.

##### 2.7.4.3 Peak Processing

XCMS software was employed for retention time correction, peak detection, extraction, integration, and alignment, with parameters set to minfrac = 0.5 and cutoff = 0.3.

##### 2.7.4.4 Lipid Identification

Lipid annotation was performed using an in‐house pipeline integrating XCMS, custom R packages, and the LipidBlast database. Metabolite identities were confirmed by matching experimental spectra to reference spectral libraries.

#### 2.7.5 Machine Learning Algorithms

Principal Component Analysis (PCA) transforms a set of possibly correlated variables into linearly uncorrelated principal components through orthogonal transformation, thereby reducing the dimensionality of the data. In this study, data were subjected to log transformation and mean centering using SIMCA software before performing automatic modeling analysis. PCA is capable of revealing the underlying structure of the data, illustrating the overall distribution trends of the samples and the degree of differences between groups, thereby providing essential information regarding the global characteristics of the dataset for subsequent analyses.

OPLS‐DA addresses the high‐dimensionality, small‐sample nature, and complex variable correlations inherent in metabolomics data. By segregating orthogonal (non‐correlated) variables from predictive components, the algorithm enhances interpretability of group‐specific metabolic differences. Model validity was assessed through cross‐validation and permutation testing in SIMCA. This supervised approach robustly identifies metabolites driving inter‐group discrimination, serving as a cornerstone for elucidating metabolic perturbations across experimental conditions.

#### 2.7.6 Statistical Analysis

Statistical analyses were performed using SPSS (version 21.0), with the chi‐square test/Fisher exact test for categorical data comparison and Wilcoxon rank‐sum test for ranked data. Applied the *t*‐test (or *t*' test for unequal variances) for quantitative data with normal distributions, while the Wilcoxon rank‐sum test for non‐normal data. Results were presented as mean ± standard deviation (mean ± SD) for normal distribution and median (range) for non‐normal distribution. A two‐sided *p* < 0.05 indicated statistical significance. Orthogonal partial least squares‐discriminant analysis (OPLS‐DA) and permutation tests were conducted in R (version 3.3.5) for selected metabolites meeting criteria of *p* < 0.05 and Variable Importance in the Projection (VIP) > 1. Differences of metabolites between groups were visualized through volcano plots, heatmaps, bar plots, and bubble plots in R. Pathway enrichment analysis was performed using the MetaboAnalyst database.

## 3 Results

### 3.1 Patient Characteristics

Eleven newly diagnosed SAA patients, including 6 females and 5 males were enrolled; the median age and BMI were 53 (22–64) years and 22.95 (18.14–23.74) kg/m^2^ respectively (Table 1). Six of these patients (equally 3 females and 3 males) received ATG‐based IST and were selected and sub‐grouped based on their 9‐month responses. The remaining 5 patients received allo‐HSCT afterward. Comparisons of baseline clinical parameters, including age, ANC, and Ret%, showed no statistically significant differences between responders and non‐responders (all *p* > 0.05), indicating the two groups were comparable at baseline. In addition, serum samples derived from PB and BM of 15 healthy donors (10 males and 5 females) were used as controls, with a comparable median age and BMI.

**TABLE 1:** The baseline characteristics of SAA patients and treatments.

PT	Gender (male/female)	Age (years)	BMI (kg/m^2^)	Initial WBC (×10^9^/L)	Initial ANC (×10^9^/L)	Initial Hb (g/L)	Initial PLT (×10^9^/L)	Ret (%)	Prior temporary treatment (less than 1 month)	Treatments in study
1	M	55	23.32	0.2	0.1	55	7	0.18	G‐CSF + rhTPO	ATG‐based IST
2	F	62	23.74	1.2	0.3	57	7	0.37	EPAG + androgen	ATG‐based IST
3	M	52	21.05	2	0.8	55	14	1.68	Androgen	ATG‐based IST
4	F	53	23.67	3.3	0.5	65	19	2.72	None	ATG‐based IST
5	F	58	23.24	0.3	0.3	56	10	3.06	None	ATG‐based IST
6	M	64	18.14	0.6	0.6	60	9	1.41	None	ATG‐based IST
7	M	28	22.95	1.1	0.1	69	10	0.21	G‐CSF	Allo‐HSCT
8	F	56	18.73	3.3	0.8	62	14	1.43	None	CsA+ androgen
9	F	53	23.53	0.1	0.1	51	4	0.27	None	Allo‐HSCT
10	F	49	19.47	0.4	0	71	3	0.10	None	Allo‐HSCT
11	M	22	21.71	2.6	0.6	48	14	1.49	None	Allo‐HSCT
All	5/6	53 (22–64)	22.95 (18.14–23.74)	1.573 ± 1.142	0.382 ± 0.293	59 ± 7.211	10.091 ± 4.826	1.2 ± 1	/	/

Abbreviations: Allo‐HSCT, allogeneic hematopoietic stem cell transplantation; ATG, anti‐thymocyte globulin; CsA, cyclosporine A; EPAG, eltrombopag; F, female; G‐CSF, granulocyte colony‐stimulating factor; IST, immunosuppressive therapy; M, male; rhTPO, recombinant human thrombopoietin.

### 3.2 ATG‐Based IST Response

Of the 6 patients receiving ATG‐based IST, the response rate was 50% (3/6) at 9 months after treatment. The median follow‐up duration for the response and non‐response groups was 12 (12–18) months and 12 (9–12) months, respectively. For the responders, 1 case achieved CR at 5 months after treatment.

### 3.3 Estimation of the OPLS‐DA Model and the Overall Difference Between SAA and Healthy Donors

**FIGURE 1:**
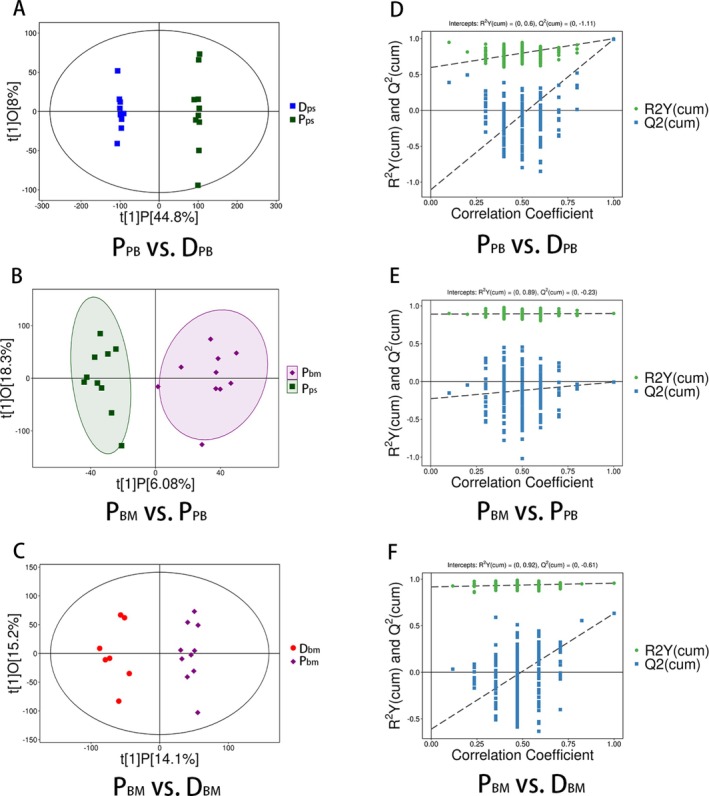
Score scatter plots (A–C) and permutation test plots (D–F) of OPLS‐DA model. (A–C) In the figure, the x‐axis t[1]P showed the predicted principal component score of the first principal component, represented the overall difference between the comparison groups, and the y‐axis t[1]O represented the orthogonal principal component scores, demonstrating the difference between samples within each group. The farther the horizontal distance between samples, the greater the difference between groups. Closer longitudinal distances indicate better within‐group reproducibility. (D–F) The x‐axis of permutation plot tests represented the correlation coefficient, the y‐axis represented R^2^Y or Q^2^, which described the interpretation level and prediction level of the model. The values of R2 and Q^2^ close to 1 indicated the model was less susceptible to overfitting. The OPLS‐DA model was considered to have no overfitting if the intercept on the Y‐axis of the Q^2^ regression line was less than 0.05. BM, bone marrow; D, donor; P, patient; PB, peripheral blood.

**FIGURE 2:**
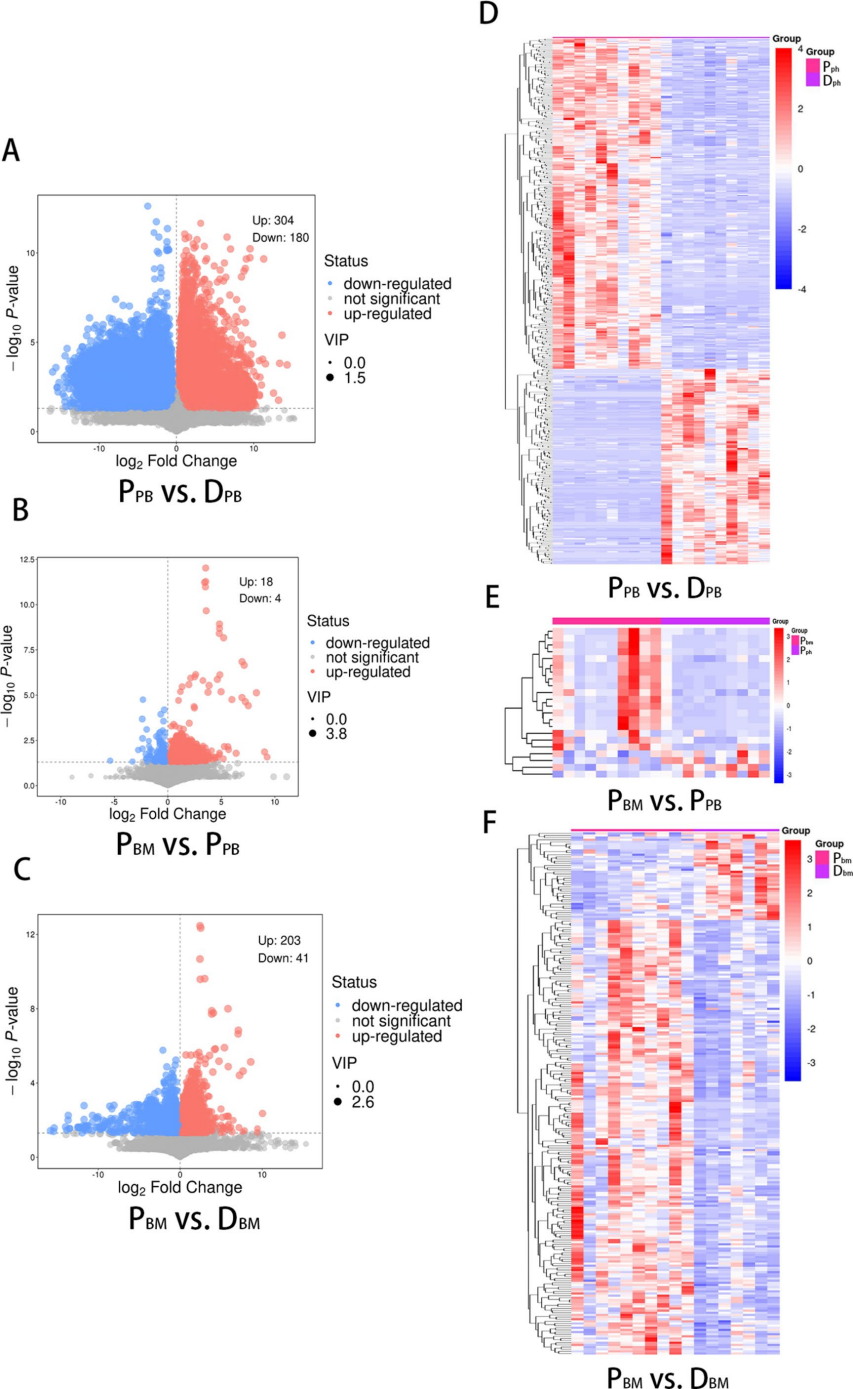
Volcano plots (A–C) and heatmaps (D–F) for differential metabolites between SAA and donors in peripheral blood and bone marrow serum before treatment. (A–C) Volcano plots visualizing the distribution of differential metabolites. The differential metabolites were visualized in the form of volcano plots. Red dots represented significantly up‐regulated metabolites, and blue dots represented significantly down‐regulated metabolites. The selection criteria for differential metabolites were a Variable Importance in the Projection (VIP) > 1 and a *p*‐value < 0.05. Gray dots represent metabolites that did not meet these criteria. (D–F) Heatmaps of the differential metabolites. The x‐axis represented the different groups, and the y‐axis represented the differential metabolites of each group. Red blocks indicated high expression of differential metabolites, while blue blocks indicated low expression. BM, bone marrow; D, donor; P, patient; PB, peripheral blood.

The score scatter plots of the OPLS‐DA model showed the differences between patients (P) and donors (D) (Figure 1A–C), and overfitting was assessed by permutation tests (Figure 1D–F). For biological samples, Q^2^ ≥ 0.4 was ideal. The OPLS‐DA model of the P_PB_ versus D_PB_ group and P_BM_ versus D_BM_ group had good accuracy, and there was no overfitting. In the P_BM_ versus P_PB_ group, Q^2^ was < 0 while the intercept on the Y‐axis of the regression line was < 0.05, indicating that the accuracy was poor, but there was no overfitting. The volcano map (Figure 2A–C) and heat map (Figure 2D–F) confirmed differences in subgroups to a certain degree.

### 3.4 Pre‐Treatment Lipid Metabolism Profiles of SAA Patients and Healthy Donors

**FIGURE 3:**
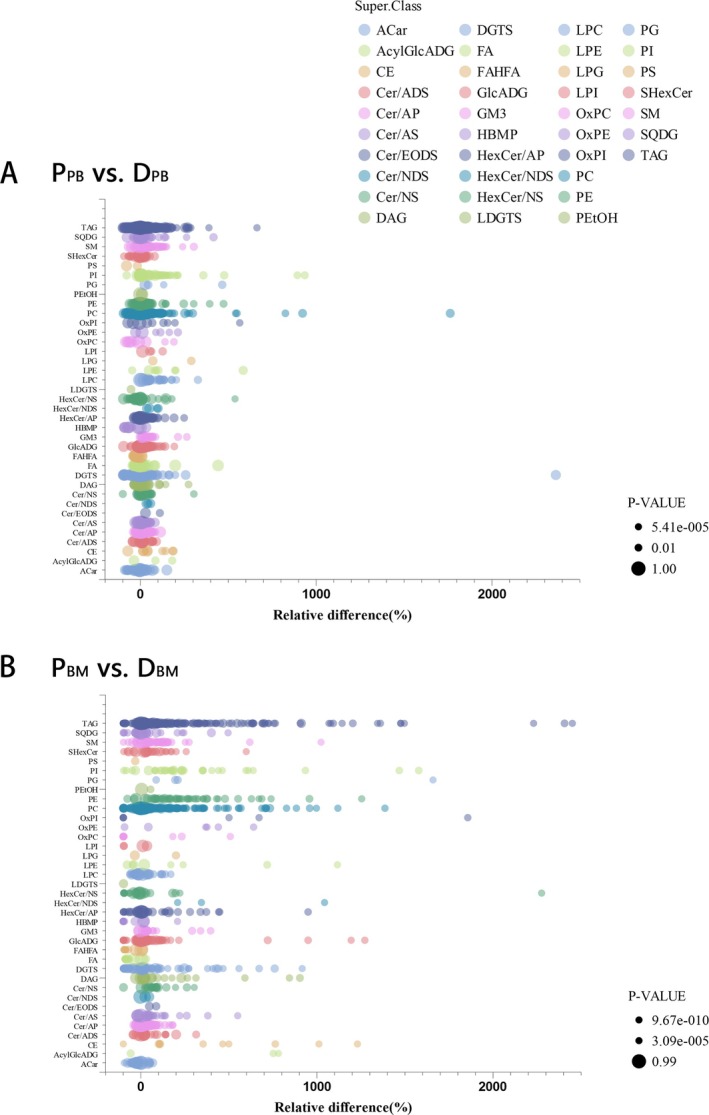
Bubble plots for comparing the peripheral blood (A) and bone marrow (B) serum between SAA and donors before treatment. The x‐axis represents the relative difference (%), which serves as a metric for the fold change of lipid metabolites between groups. It is calculated using the formula: Relative Difference (%) = (Mean_Patient_—Mean_Donor_)/Mean_Donor_ × 100. Consequently, a positive value indicates that the corresponding metabolite corresponds to a higher abundance in the patient group P_PB_ and P_BM_, while a negative value indicates a lower abundance compared to donors. The y‐axis represents the specific lipid species. Bubble size reflects the statistical significance (*p*‐value). BM, bone marrow; D, donor; P, patient; PB, peripheral blood.

**FIGURE 4:**
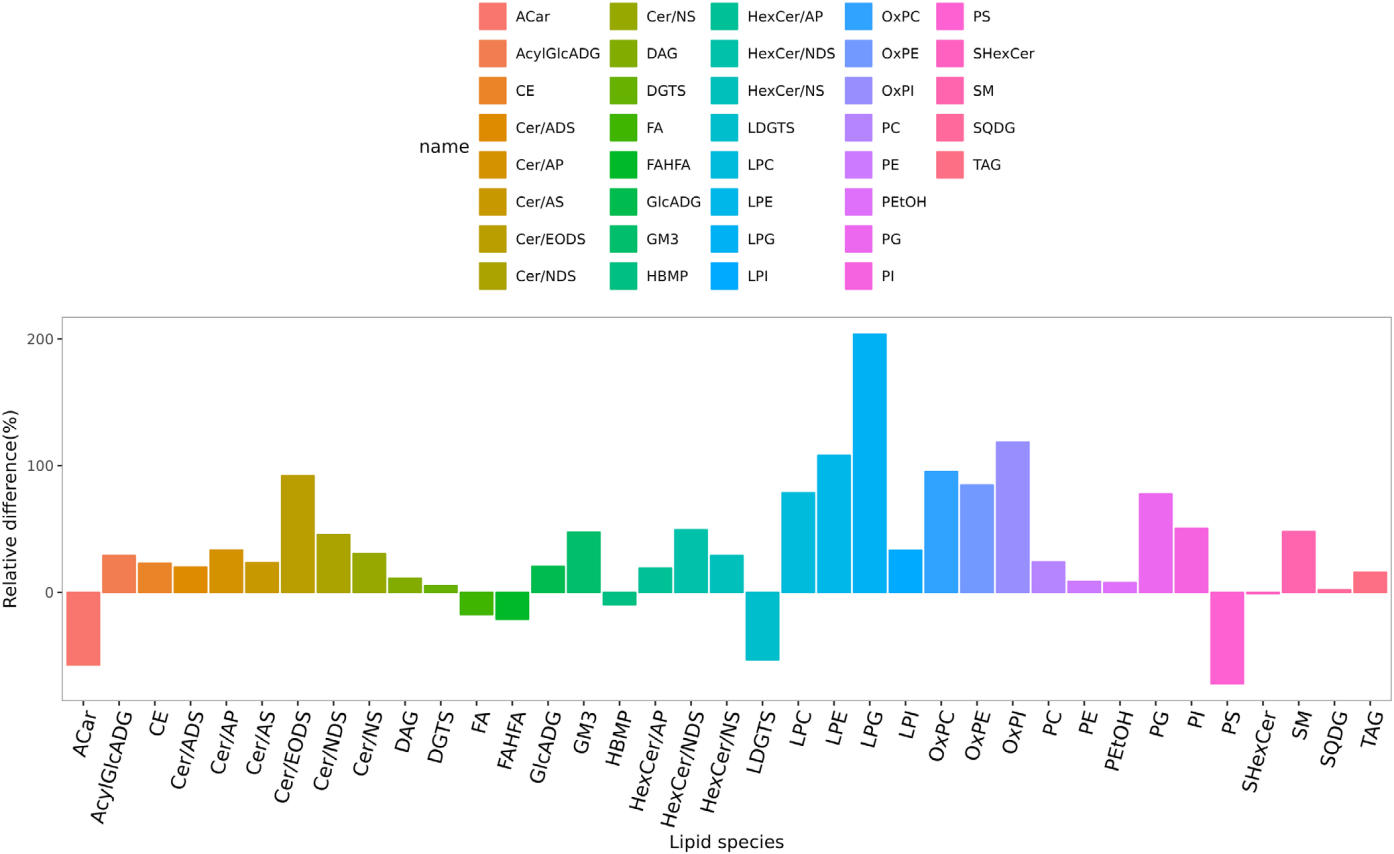
Bar plots for comparing the bone marrow serum between SAA and donors before treatment. In the comparison of lipid metabolism profiles in bone marrow serum of SAA patients and donors, most of the metabolites showed positive relative differences, indicating that these metabolites were more abundant in the P_BM_ group. BM, bone marrow; D, donor; FAHFA, fatty acid ester of hydroxyl fatty acid; HexCer‐NS, hexosylceramide non‐hydroxy fatty acid‐sphingosine; P, patient. A complete list of lipid abbreviations and full names is provided in Table S1.

**FIGURE 5:**
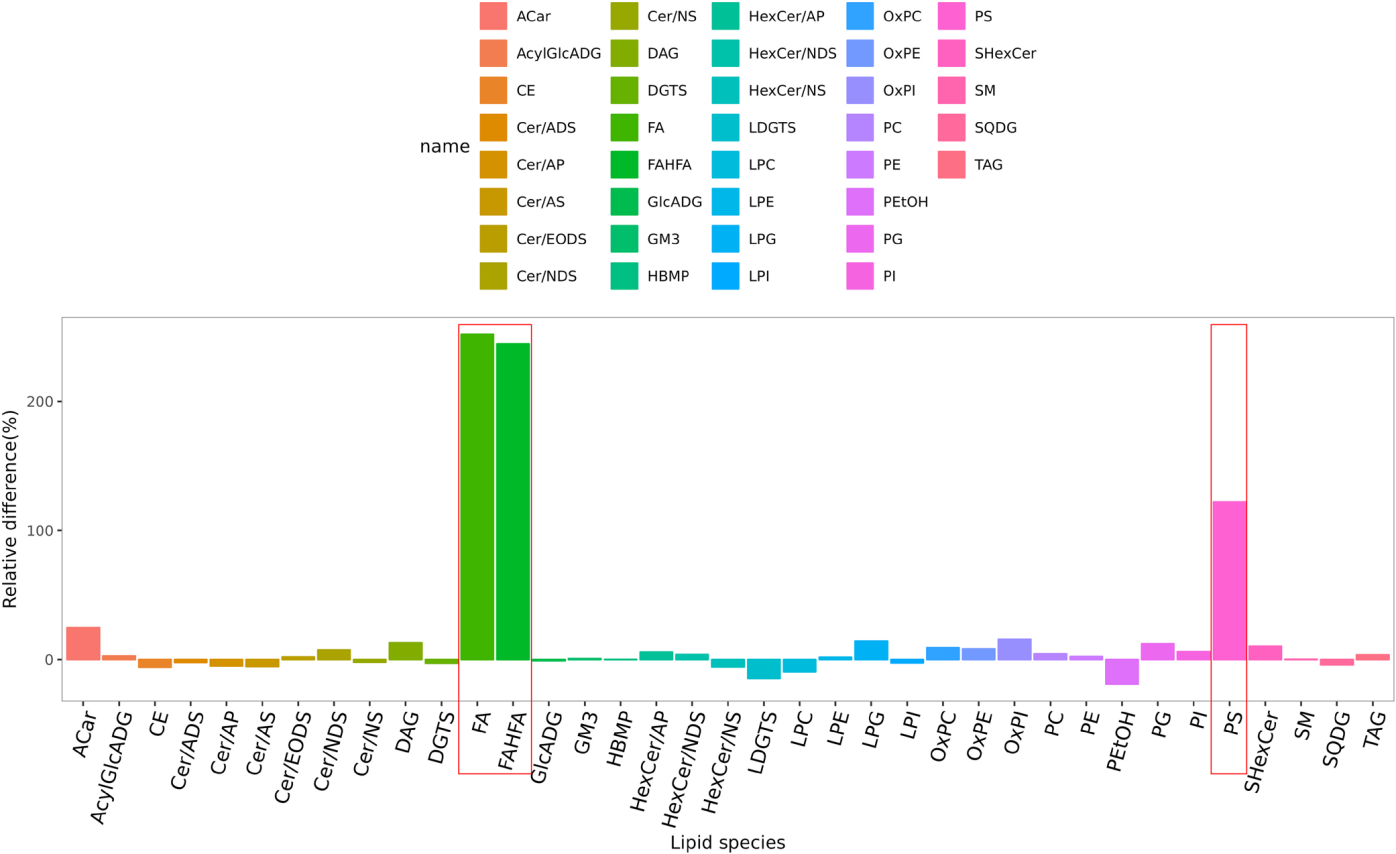
Bar plots for comparing the peripheral blood and bone marrow serum in SAA patients before treatment. In the comparison of lipid metabolism profiles between bone marrow serum and peripheral blood serum of SAA patients, the free fatty acid (FA) and fatty acid ester of hydroxyl fatty acid (FAHFA), phosphatidylserine (PS) selected in red box had an obvious relative difference compared with other differential lipid metabolites. BM, bone marrow; D, donor; P, patient; PB, peripheral blood. A complete list of lipid abbreviations and full names is provided in Table S1.

The baseline lipid metabolism profiles were compared between patients (P) and healthy donors (D) in PB serum (P_PB_ vs. D_PB_) (Figure 3A) and BM serum (P_BM_ vs. D_BM_) (Figure 3B), respectively. Notably, the differences between patients and donors were more pronounced in BM serum than in PB serum. Moreover, the lipid metabolism profiles of BM demonstrate diminished responsiveness to extrinsic factors, including dietary intake, endocrine fluctuations, and other environmental influences, thereby indicating that BM serum may serve as a more reliable clinical sample for research purposes. Analysis of baseline BM serum revealed a pronounced enrichment of the majority metabolites in patients when compared to donors (P_BM_ vs. D_BM_), suggesting the presence of dysregulated lipid metabolism in SAA patients (Figure 4). Furthermore, a comparative analysis of baseline BM and PB serum in patients was conducted (P_BM_ vs. P_PB_) (Figure 5), which demonstrated a significant up‐regulation of free fatty acids (FA), fatty acid ester of hydroxyl fatty acid (FAHFA), and phosphatidylserine (PS) in BM serum.

### 3.5 Changes of Lipid Metabolism Profiles After Treatment

**FIGURE 6:**
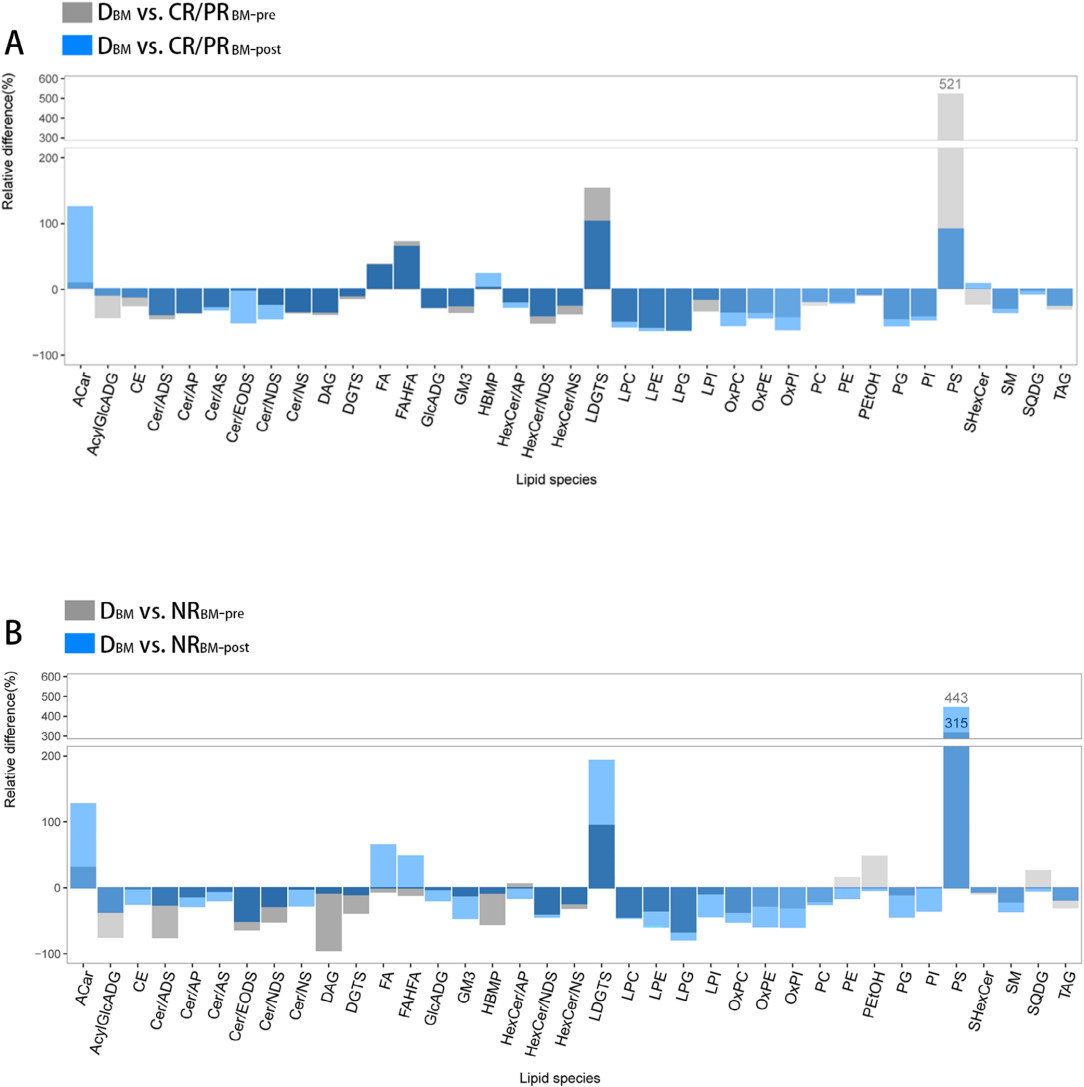
Bar plots for comparing the lipid metabolism profiles in bone marrow serum from donors and patients with complete or partial response (A) and no response (B) pre‐ and post‐treatment. The y‐axis displays the relative difference (%), representing the percentage change of lipid abundance in patient subgroups relative to the healthy donor baseline (D_BM_). This was calculated as: (Mean_Subgroup_—Mean_Donor_)/Mean_Donor_ × 100. Gray bars represent pre‐treatment comparisons (D_BM_ vs. CR/PR_BM‐pre_), and blue bars represent post‐treatment comparisons (D_BM_ vs. CR/PR_BM‐post_). Positive values indicate upregulation, and negative values indicate downregulation compared to healthy donors. BM, bone marrow; CR, complete response; D, donor; P, patient; PR, partial response. A complete list of lipid abbreviations and full names is provided in Table S1.

Comparative analyses of lipid metabolism profiles in BM serum from donors and patients with complete or partial response (Figure 6A) and no response (Figure 6B) pre‐ and post‐treatment (D_BM_ vs. CR/PR_BM‐pre_ and D_BM_ vs. CR/PR_BM‐post_, D_BM_ vs. NR_BM‐pre_, D_BM_ vs. NR_BM‐post_) revealed that the relative difference of acylcarnitine (ACar) and hexosylceramide non‐hydroxy fatty acid‐sphingosine (HexCer‐NS), sulfur hexosylceramide hydroxy fatty acid (SHexCer) up‐regulated after IST, with more pronounced changes observed in CR/PR patients compared to NR patients. This result suggested that these metabolites may play a protective role in the context of immune reprogramming. Conversely, lysophosphatidylcholine (LPC) and oxidized phosphatidylcholine (OxPC) were down‐regulated after IST, with more substantial alterations observed in CR/PR patients compared to NR individuals. This finding indicated that these metabolites may hinder the recovery of hematopoietic function or may be significantly depleted during the process of hematopoietic recovery.

### 3.6 Pathways Enrichment Analysis

**FIGURE 7:**
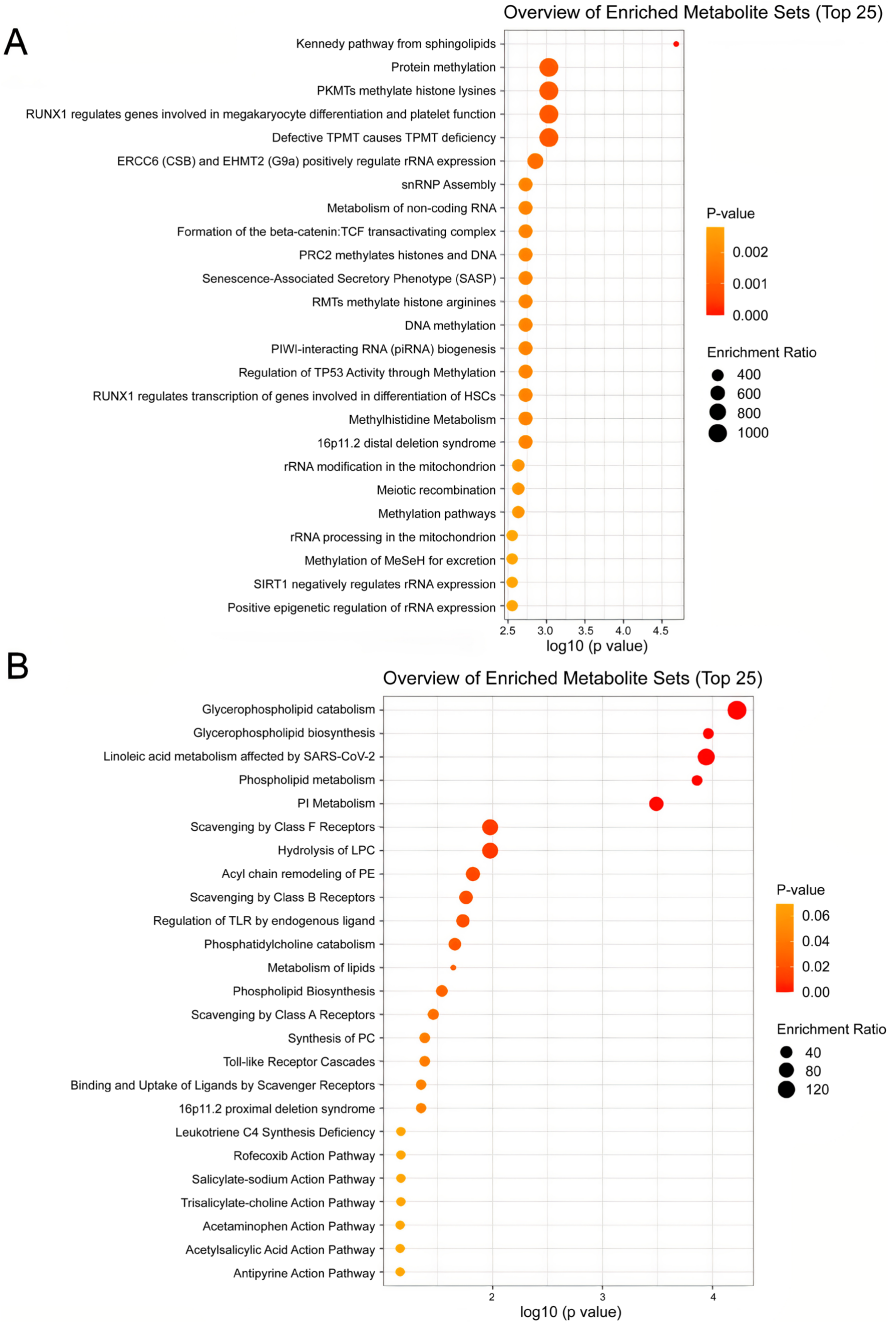
Bubble plots of lipid metabolism pathway enrichment analysis. (A) The lipid metabolism pathway enrichment analysis result of up‐regulated metabolites after IST. The y‐axis represented the corresponding pathway names and the x‐axis showed −log10 (*p* value). The bubble size reflected the enrichment ratio. (B) The lipid metabolism pathway enrichment analysis result of down‐regulated metabolites after IST. The y‐axis, x‐axis, and bubble size maintained the same definitions as described above.

Pathway enrichment analysis conducted via MetaboAnalyst revealed that the up‐regulated differential metabolites after IST were associated with various metabolic pathways (Figure 7A). These metabolites were particularly linked to the Kennedy pathway from sphingolipid metabolism, RUNX1 regulates genes involved in megakaryocyte differentiation and platelet function, and RUNX1 regulates transcription of genes involved in the differentiation of HSCs. In contrast, the down‐regulated differential metabolites post‐IST were predominantly associated with the metabolism of glycerophospholipids, linoleic acid, phospholipids, and specific classes including phosphatidylinositol (PI), lysophosphatidylcholine (LPC), PE, and PC. These findings imply that IST may play a role in the restoration of BM homeostasis by modulating these metabolic pathways (Figure 7B).

## 4 Discussion

In the comparative analysis of lipidomic profiles between patients and healthy individuals, the observed differences in BM were more pronounced. Lipid metabolism in the BM serves not only as a source of energy but also plays a critical role in modulating hematopoiesis. This modulation occurs through the constitution and influence of various metabolic pathways, imparting both positive and negative effects on hematopoietic processes (Pernes et al. 2019). Lipid metabolism was reported to be associated with the pathogenesis of abnormal hematopoiesis. Prior research has demonstrated that dysregulated function, quantity, and differentiation of MSCs contribute to boosting adipocytes (Wei et al. 2018). Adipocyte‐derived factors, such as leptin, create a potent proinflammatory environment for T cells, which eventually led to the onset and progression of AA (Gao et al. 2022). Reprogramming of lipid metabolism, especially the metabolic pathways associated with fatty acid, cholesterol, and phospholipid, has been identified as a pivotal component of the carcinogenic mechanisms underlying hematological malignancies. This dysregulation is associated with an adverse prognosis, highlighting its significance in disease outcomes (Zhang et al. 2022).

Given the current lack of comprehensive research focusing on the alterations in lipid metabolism among patients with SAA, the mechanisms underlying clinical response improvement, the identification of potential adverse prognostic factors, and the optimization of future therapeutic strategies remain unclear. These aspects are essential for enhancing the overall prognosis of individuals afflicted with SAA. Analysis of the relative difference between BM serum and PB serum in SAA patients at baseline in this study revealed that fatty acids were significantly up‐regulated in BM compared with other differential metabolites. This observation suggests a potential hyperproduction or transportation dysregulation of fatty acids in BM relative to PB serum, which is consistent with the previous literature (Wang et al. 2022). Within the context of fatty acid metabolism, arachidonic acid metabolism was considered to be related to the occurrence of AA early in 1990 (Das 1990). Additionally, Ruan J et al. (Ruan et al. 2023) also suggested that the arachidonic acid metabolites PGE2, 15(S)‐HETE, and LTB4 regulate hematopoiesis through distinct mechanisms. PGE2 and PGD2 stimulate proliferation and differentiation of erythroid progenitor cells, while 15(S)‐HETE directs erythroid lineage commitment and LTB4 promotes clonal expansion of myeloid progenitor cells. Patients with AA exhibit significantly reduced activity in this metabolic pathway, contributing to bone marrow failure.

In this study, the implementation of IST resulted in significant alterations to lipid metabolism. Comparative analyses of differential metabolites before and after treatment in SAA patients revealed that ACar, HexCer‐NS, and SHexCer were up‐regulated after IST, with a more pronounced increase observed in CR/PR patients. The low baseline levels of these metabolites may be associated with metabolic impairments or the presence of antibodies. Research has pointed out that changes in the relative concentration of ACar, an intermediate lipid metabolite within fatty acid metabolic pathways, serve as markers of fatty acid metabolism damage (Yamakawa et al. 2023). Halmer R et al. demonstrated a significant up‐regulation of antibodies against SHexCer in patients with multiple sclerosis (MS), who frequently present with complex autoimmune disorders, thereby suggesting a possible relationship between increased anti‐SHexCer antibodies and autoimmune pathogenesis (Halmer et al. 2014). As a specific subtype of hexosylceramides (HexCer), HexCer‐NS currently has not been extensively studied in the field of AA. Previous investigations indicated that HexCer levels were consistently elevated in some patients with large granular leukocyte (LGL) leukemia (Olson et al. 2020). Given that LGL leukemia patients often have concurrent autoimmune diseases (Lamy et al. 2017), the abnormal accumulation of HexCer in SAA patients may reflect a degree of immune dysregulation. Existing literature has suggested that sphingolipids may negatively affect the maintenance of HSCs stemness. However, recent research has confirmed that inhibiting the DEGS1 gene leads to the accumulation of specific sphingolipids, which, in turn, helps sustain HSCs stemness (Xie et al. 2019). This implies that IST may enhance hematopoietic function in the BM of SAA patients by modulating sphingolipid metabolism. Furthermore, the results of this study indicate that LPC and OxPC were down‐regulated after IST. Investigations into LPC in various human diseases have primarily focused on atherosclerosis, cardiovascular diseases, and neurodegenerative disorders, wherein LPC exacerbates disease progression by compromising endothelial cell integrity and disrupting endothelial barrier function. Although direct research on LPC within the field of hematology remains limited, the findings of this study may offer valuable insights into potential adverse prognostic factors and enhance our understanding of disease progression in SAA (Halmer et al. 2014, Law et al. 2019).

IST may influence hematopoietic reconstruction and functional recovery by modulating sphingolipid metabolism, as well as megakaryocyte and platelet function and differentiation of HSCs. The identified differential metabolites, alongside their associated metabolic pathways, provide novel insights into strategies aimed at enhancing the stability and functionality of the hematopoietic system. Moreover, the related pathways primarily govern gene expression and cellular functions via mechanisms such as DNA methylation, histone modifications, and non‐coding RNA regulation. This suggests that IST may exert its regulatory effects on cellular development, functional maintenance, and disease outcomes through intricate epigenetic mechanisms.

Further pathway enrichment analysis of down‐regulated differential metabolites suggests that IST might be associated with the restoration of homeostasis within the BM microenvironment, potentially involving alterations in the metabolism of glycerophospholipids, linoleic acid, phospholipids, PI, LPC, PE, and PC. While a study conducted in 1987 confirmed the stimulatory effect of linoleic acid on hematopoiesis (Young et al. 1987), the current findings of this study imply that linoleic acid may exert a complex and bidirectional impact on hematopoietic processes in this context. PI is characterized as an upstream regulator crucial for maintaining Hippo pathway activity (Li et al. 2022). Given that the Hippo pathway regulates adipocyte proliferation and differentiation (Huang et al. 2013) by potentially suppressing peroxisome proliferator‐activated receptor‐γ (PPARγ), a key player in adipogenesis (Hong et al. 2005), we speculate that the differential PI levels observed in this study could theoretically influence these regulatory axes. Previous studies focusing on glycerophospholipids and OxPC have been mainly linked to neurodegenerative conditions (Dong et al. 2021, Frisardi et al. 2011), and, to date, there is a paucity of data elucidating their role in the pathophysiology of AA.

### 4.1 Further Clinical Applications

Existing research indicates that quantitative analysis of ACar can activate antitumor immune responses by enhancing mitochondrial oxidative phosphorylation in B cells, inducing epigenetic reprogramming and activating the PD1 protein degradation pathway, thereby providing a foundation for a precise “detection‐intervention‐reassessment” treatment cycle (Zhang et al. 2024). The Acetylcarnitine (ALCAR) ELISA Kit (Abbexa, catalog no. abx258336) has been utilized for precise quantification of ALCAR levels, a specific molecular species within the ACar family. Meanwhile, commercialized LPC and OxPC detection kits (US Biological, catalog no. 348284 and Aanti Polar Lipids, catalog no. 330600) have been used to assess lipid levels in samples, and the widespread use of these kits has achieved high‐throughput and precise quantification.

Although HexCer‐NS and SHexCer do not yet have mature commercialized kits, they can be detected by optimizing ELISA technology. In the future, as these detection technologies continue to develop and expand, they will provide dynamic monitoring support for patients' lipid metabolism profiles, thereby optimizing precision treatment strategies and providing critical molecular evidence for the construction of prognostic prediction models.

Building upon current research findings, we will conduct multicenter clinical validation to further confirm the clinical value of ACar, LPC, and OxPC as predictive biomarkers for IST, while establishing standardized detection protocols for HexCer‐NS/SHexCer.

### 4.2 Study Strengths and Limitations

This study supplemented the changes in BM lipid metabolism in AA patients throughout the treatment of ATG‐based IST, revealing its significance in the process of ATG‐based IST treatment. However, many limitations remain in this work; a larger sample size is needed to verify the conclusions, and further specific indexes deserve further validating for the earlier IST response prediction.

### 4.3 Conclusion

Acknowledge of the changes of bone marrow lipidomics can contribute to earlier prediction of ATG‐based IST response in SAA patients. The metabolites ACar, HexCer‐NS, and SHexCer may help restore lipid metabolic homeostasis in the BM during IST. Conversely, LPC and OxPC may function as detrimental factors in metabolic processes or they may be depleted during the recovery of hematopoietic function. Consequently, optimizing the levels of these metabolites may be a potential strategy for enhancing hematopoietic recovery in SAA patients.

## Author Contributions

D.W. conceived and designed the study. Z.S., Y.H, C.X. and Q.L. assisted in the acquisition of data. Z.S., Y.H., Y.C., and S.L. analyzed and interpreted the data. Z.S. and D.W. wrote, reviewed, and revised the manuscript. H.H., Y.S., Y.Z., and D.W. took care and followed up patients. All authors contributed toward data analysis, drafting, and critically revising the paper and agreed to be accountable for all aspects of the work.

## Funding

The present study was supported by the National Key R&D Program of China (Young Scientist Project) (NO. 2025YFC3511400), National Natural Science Foundation of China (NO. 82174138, NO. 82304937), Zhejiang Scientific Research Fund of Traditional Chinese Medicine (NO. 2020ZB085), Health Technology Plan of Zhejiang Province (NO. 2022RC216), and Project of Zhejiang Famous Traditional Chinese Medicine Expert Inheritance Studio Construction (NO. GZS2021022).

## Disclosure

The authors have nothing to report.

## Ethics Statement

This study received approval from the ethical committee of the First Affiliated Hospital of Zhejiang Chinese Medical University (NO. 2022‐KL‐057‐01). All procedures conducted involving human participants adhered to the ethical standards set forth by the institutional and/or national research committee, in compliance with the 1964 Helsinki Declaration and its subsequent amendments, or comparable ethical standards. Informed consent was obtained from all participating patients prior to their inclusion in the study.

## Conflicts of Interest

The authors declare no conflicts of interest.

## Supporting information


**Figure S1:** PCA score plot of lipid metabolic profiles across all study groups and quality control (QC) samples.
The tight clustering of QC samples (Cyan diamonds) confirms the high stability and reproducibility of the LC–MS system throughout the analytical run. D_BM_ (Red circles): Donor Bone Marrow; D_PB_ (Blue squares): Donor Peripheral Blood; P_BM_ (Purple diamonds): Patient Bone Marrow (Baseline/Pre‐treatment); P_BM‐post_ (Orange circles): Patient Bone Marrow (Post‐treatment); P_PB_ (Dark Green squares): Patient Peripheral Blood; PC (Cyan diamonds): QC samples.

**Table S1:** A complete list of lipid abbreviations and full names.

## Data Availability

The data that support the findings of this study are available on request from the corresponding author. The data are not publicly available due to privacy or ethical restrictions.
