# Supplementary figures and images for: Peripheral versus Marrow Lipidomics in Patients with Severe Aplastic Anemia: Potential Indicators for Early Immunosuppressive Treatment Response
Source: Lipids. 2026 Jan 19;61(3):347–61. doi: 10.1002/lipd.70034 (PMC13144709; doi:10.1002/lipd.70034)

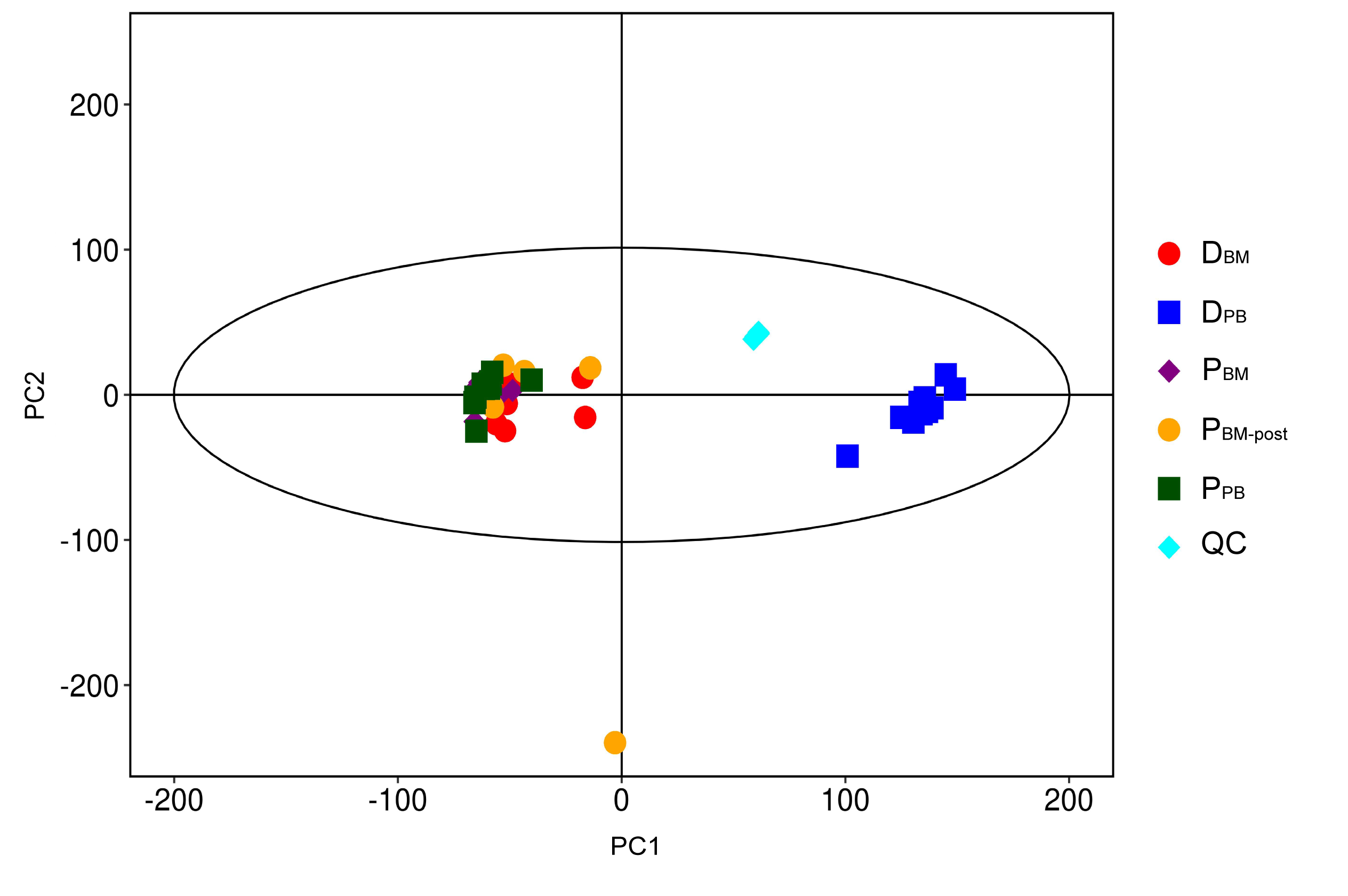

Supplement: Supplementary file 1 — Figure S1: PCA score plot of lipid metabolic profiles across all study groups and quality control (QC) samples. The tight clustering of QC samples (Cyan diamonds) confirms the high stability and reproducibility of the LC–MS system throughout the analytical run. DBM (Red circles): Donor Bone Marrow; DPB (Blue squares): Donor Peripheral Blood; PBM (Purple diamonds): Patient Bone Marrow (Baseline/Pre‐treatment); PBM‐post (Orange circles): Patient Bone Marrow (Post‐treatment); PPB (Dark Green squares): Patient Peripheral Blood; PC (Cyan diamonds): QC samples. [file LIPD-61-347-s001.tif]
